# Distinct In Vitro Effects of Liposomal and Nanostructured Lipid Nanoformulations with Entrapped Acidic and Neutral Doxorubicin on B16-F10 Melanoma and Walker 256 Carcinoma Cells

**DOI:** 10.3390/pharmaceutics17070904

**Published:** 2025-07-12

**Authors:** Roxana Pop, Mădălina Nistor, Carmen Socaciu, Mihai Cenariu, Flaviu Tăbăran, Dumitriţa Rugină, Adela Pintea, Mihai Adrian Socaciu

**Affiliations:** 1 Faculty of Veterinary Medicine, University of Agricultural Sciences and Veterinary Medicine, 400372 Cluj-Napoca, Romania; roxana.pop@usamvcluj.ro (R.P.); nistor.madalina@usamvcluj.ro (M.N.); carmen.socaciu@usamvcluj.ro (C.S.); mihai.cenariu@usamvcluj.ro (M.C.); alexandru.tabaran@usamvcluj.ro (F.T.); apintea@usamvcluj.ro (A.P.); 2 Department of Biotechnology, Biodiatech—Research Centre for Applied Biotechnology in Diagnosis and Molecular Therapy, 400478 Cluj-Napoca, Romania; mihai.socaciu@umfcluj.ro; 3 Faculty of Medicine, University of Medicine and Pharmacy “Iuliu Haţieganu”, 400347 Cluj-Napoca, Romania

**Keywords:** doxorubicin, liposomes, nanolipid, carriers, melanoma, mammary, cells

## Abstract

**Background:** Liposomes and, more recently, structured nanolipid particles have demonstrated effectiveness as carriers for delivering hydrophilic or lipophilic anticancer agents, enhancing their biocompatibility, bioavailability, and sustained release to target cells. **Objective:** Herein, four doxorubicin formulations—comprising either the acidic or neutral form—were encapsulated into liposomes (Lipo) or nanostructured lipid carriers (NLCs) and characterized in terms of size, entrapment efficiency, morphology, and effects on two cancer cell lines (melanoma B16-F10 and breast carcinoma Walker 256 cells). **Methods and Results:** While liposomal formulations containing acidic doxorubicin displayed IC_50_ values ranging from 1.33 to 0.37 µM, NLC-based formulations, particularly NLC-Doxo@Ac, demonstrated enhanced cytotoxicity with IC_50_ values as low as 0.58 µM. Neutral Doxorubicin demonstrated lower cytotoxicity in both the nanoformulations and cell lines. Differences were also observed in their internalization patterns, cell-cycle impact, and apoptotic/necrotic effects. Compared to liposomes, NLCs exhibited distinct internalization patterns and induced stronger cell-cycle arrest and necrosis, especially in melanoma cells. Notably, NLC-Doxo@Ac outperformed liposomal counterparts in melanoma cells, while liposomal formulations showed slightly higher efficacy in Walker cells. Early and late apoptosis were more pronounced in Walker cells, whereas necrosis was more prominent in melanoma B16-F10 cells, particularly with the nanolipid formulations. **Conclusions:** These results correlated positively with cell-cycle measurements, highlighting the potential of NLCs as an alternative to liposomes for the delivery of neutral or acidic doxorubicin, particularly in tumor types that respond poorly to conventional formulations.

## 1. Introduction

Over the past few decades, a wide range of nanoparticle-based drug delivery systems have been thoroughly developed and investigated for cancer therapy. Among these, lipid-based nanoparticles, such as liposomes, solid lipid nanoparticles (SLNs), phytosomes, nanoemulsions, nanostructured lipid carriers (NLCs), lipid-coated nanoparticles, and nano-lipoproteins, have been the focus of ongoing review and refinement in terms of their formulation, characterization, and application in various drug delivery approaches [1,2,3,4,5,6]. Compared to free anticancer drugs, lipid-based nanoparticles with incorporated drugs have many advantages, such as improved stability, permeability and retention, biocompatibility, and more precise targeting. The best-known nanodelivery vehicles are spherical liposomes, consisting of phospholipid bilayers with a hydrophilic core, which can incorporate mainly hydrophilic drugs and small lipophilic drugs within the membrane [7,8,9,10].

Doxorubicin (Doxo), a cytotoxic anthracycline antibiotic, is one of the most widely used anticancer DNA damage-inducing drugs, and one of the most efficient chemotherapeutics recommended by the Food and Drug Administration (FDA) for the treatment of melanoma, breast and lung cancers, hepatocarcinomas, leukemia, and malignant lymphoma [11,12,13]. However, its therapeutic effectiveness is often accompanied by significant side effects, as its mechanism actions, such as DNA intercalation, inhibition of topoisomerase II, and generation of reactive oxygen species, can lead to DNA damage and subsequent cell death. Additionally, it may lead to cardiotoxicity and the suppression of blood cell production. Meanwhile, Doxo inhibits the growth and division of cancer cells and induces apoptosis [14,15,16]. Therefore, different nanoformulations, especially liposomal ones, have shown the enhanced effectiveness of Doxo through improved delivery, reduced side effects, and decreased drug resistance [11,17]. Recently, the potential of liposomes and polymeric micelles to enhance Doxo accumulation within tumor tissues [11] and improve its efficacy and safety was investigated, achieving an enhanced permeation and retention (EPR) effect against various cancers [17].

Liposomes showed, in many experiments, enhanced efficacy and reduced toxicity of anticancer drugs, such as Doxo, showing higher bioavailability, improved anticancer activity, and reduced cardiotoxicity compared to the free molecule, making these formulations of great interest in targeted medicine [13,18,19,20,21]. New versions with prolonged circulation and higher efficiency, such as PEGylated (stealth) liposomal Doxo, have been obtained [22]; some are commercially available, such as Doxil^®^ (also named Caelyx, authorized by the European Medicines Agency), for treating breast and ovarian cancers, multiple myeloma, and Kaposi’s sarcoma [23,24].

Different Doxo or Doxo-conjugates have been used to develop nanoformulations, such as liposomes, nanolipostructured carriers, polymersomes, micelles, dendrimers, polyethylene glycol-encapsulated iron oxide, and nanogels, using new incorporation techniques with higher yields in various experimental systems aimed at overcoming Doxo adverse reactions [7,23,25,26]. Different pH-sensitive Doxo–fatty acid prodrugs, having sizes of around 150 nm, remained intact in blood circulation while releasing Doxo within cancer cells with high loading efficiency and minimal side effects, influenced by the length of the fatty acid [27].

Nanostructured lipid carriers (NLCs) represent a new generation of nanolipids alongside solid lipid nanoparticles (SLNs). They include a blend of solid and liquid lipids, which form a partially crystallized system that exhibits improved stability and the ability to incorporate lipophilic drugs. New prototypes with increased bioavailability of lipophilic drugs, enhanced drug loading, improved stability, drug release, and enhanced targeting efficiency were built using different ingredients and techniques such as melt-emulsification and ultrasonication [2,4,5,6,28,29,30]. There is a paucity of data about nano-formulations including Doxo in NLCs; the most relevant is the report regarding enhanced in vitro activity and overcoming drug resistance in MCF-7/Adr cells [30].

Doxo is currently used in clinics in a hydrochlorinated form (Doxo@Ac). The basic form of Doxo (Doxo@Ne) is hydrophobic and can be used in neutral and slightly alkaline environments. However, it is not clear whether the salt form of doxorubicin influences its anticancer effect. One critical aim of recent research has been to identify the different behaviors of the same drug with varying levels of hydrophobicity in vitro and in vivo [25].

The two forms of Doxo appear to have activity dependent on pH and pKa values—implicitly on their polarity and solubility. A critical factor in the response of cancer cells to chemotherapeutic drugs is the activation of the apoptotic pathway, which is often impaired in chemoresistant melanoma or breast cancer. The incorporation of chemotherapeutic drugs into different carriers and their targeted release with local apoptotic effects on cancer cells are key bottlenecks and represent a starting point for current investigations. The hydrophilicity of the drug, the pH of the environment, and the entrapment efficiency are key factors that may influence their efficacy in vitro. Based on the acquired experience of our team [29,31,32] in preparing and characterizing liposomes and NLCs for the intracellular delivery of different bioactive molecules, we investigated the biological behavior of the neutral, lipophilic form of doxorubicin (Doxo@Ne). Both Doxo@Ne and the conventional acidic, hydrophilic form (Doxo@Ac) were encapsulated into liposomes, which served as a reference platform, and compared to their encapsulation in nanostructured lipid carriers (NLCs)—a theoretically more suitable system for delivering lipophilic drugs. The resulting formulations were characterized in terms of size, entrapment efficiency, morphology, and their effects on two cancer cell lines (melanoma B16-F10 and breast carcinoma Walker 256 cells). The impact on cell viability and toxicity, their internalization, and their effects on cell-cycle and apoptotic/necrotic processes were evaluated. Data reported here may fill an important gap in the current literature by systematically evaluating the intracellular uptake and biological effects of both acidic and neutral forms of doxorubicin delivered via NLCs, a more recent and theoretically superior delivery platform for lipophilic drug molecules.

## 2. Materials and Methods

### 2.1. Preparation of PEGylated Lipo-Doxo@Ac and Lipo-Doxo@Ne

Doxorubicin hydrochloride (Doxo@Ac) 2 mg/mL (3.45 mM) saline solution was purchased as a commercial perfusion solution from Accord-UK Ltd. (Barnstaple, United Kingdom). This solution was diluted to a final concentration of 2 mM at a pH of 7.2 using a mixture of ethanol:DMSO (3:1). To obtain the neutral Doxo-based Doxo@Ne, the method of Tahir [33] was adapted. Briefly, to a volume of 5 mL Doxo@Ac (2 mg/mL), 5 mL NaHCO_3_ solution (0.2 M) was added. After mixing (pH 8.5), the neutral, hydrophobic fraction was extracted in chloroform, concentrated under vacuum, and re-extracted in 5 mL ethanol:DMSO solution (3:1). DMSO (at final concentrations less than 1%) was chosen due to its ability to act as a cell membrane permeation enhancer in vitro and also for increasing neutral Doxo loading in the internal hydrophilic core of liposomes [1]. The conversion yield of Doxo@Ac to Doxo@Ne was determined. The final concentration of Doxo@Ne, determined by absorption at 480 nm, was 2 mM, similar to that of Doxo@Ac. Both solutions were checked for purity by HPLC with diode-array detection at 480 nm.

The procedure applied to obtain PEGylated Lipo-Doxo@Ac and Lipo-Doxo@Ne used the ethanol injection method, adapted from that of Stano et al. [34]. A total of 16 mL soybean lecithin (Sigma-Aldrich, St. Louis, MO, USA), 6.25% and 25 mg of cholesterol dissolved in pure ethanol (HPLC grade) were used in the lipid phase (LP). In the aqueous phase (AcP), 40 mL of phosphate buffer (0.01 M, pH 6.5), 40 mL of Tween 80, and 20 mg of PEG2000 were included. The AcP was heated in a water bath at 55 °C under magnetic stirring. Separately, the LP was heated at 65 °C and added dropwise to the AcP over approximately 15 min. The resulting suspension was then stirred for an additional 15 min to facilitate ethanol evaporation. Finally, the suspension was ultrasonicated at high amplitude using an UP50H Compact Lab Homogenizer (Hielscher Ultrasonics, Teltow, Germany). Empty liposomes (Control) were prepared using this procedure.

To obtain Lipo-Doxo@Ac and Lipo-Doxo@Ne, a volume of 5 mL of Doxo@Ac (2 mM) was mixed with 35 mL of AcP; following the previously mentioned procedure, 16 mL LP was added, resulting in a 40 mL liposomal suspension with a theoretical concentration of 0.25 mM Doxo@Ac. A volume of 5 mL Doxo@Ne (2 mM) was added to 16 mL LP; following the same procedure, the LP was added to 35 mL AcP, resulting in a final volume of 55 mL liposomal suspension. Therefore, the theoretical concentration of Doxo@Ne was 0.25 mM. The final concentrations of both Doxo@Ac and Doxo@Ne in the liposomes were calculated after determining the entrapment rate, as described below. Each procedure was repeated in triplicate.

### 2.2. Preparation of NLC-Doxo@Ac and NLC-Doxo@Ne

To obtain the NLC formulations NLC-Doxo@Ac and NLC-Doxo@Ne, the main ingredient used was Compritol 888 ATO, a standardized glycerol dibehenate purchased from Gattefossé (Saint-Priest, France), as previously reported for similar formulations [6,28]. The hot emulsification procedure was applied and included the addition of a volume of 50 mL hot ultrapure water phase (pH 7.2) to 2 g melted mix (at 80 °C) of Compritol, stearic acid, oleic acid, Tween 80 and Triethanolamine, at a ratio of 10:5:5:2.5:2.5 (*w*/*w*), dropwise, under ultraturax, at 20,000 rpm for 15 min. The hot emulsion was ultrasonicated, using the same UP50H Homogenizer (Hielscher), for 5 min at the highest amplitude and then kept on ice for 15 min. The resulting suspension (50 mL) contained free NLC (Control). To obtain NLC-Doxo formulations, 5 mL of Doxo@Ac (pH = 7.2) or Doxo@Ne solutions (pH = 8.5) (2 mM each) were included in 50 mL hot water phase, with their theoretical concentration being 0.2 mM. In this case, the real concentrations of both Doxo@Ac and Doxo@Ne into the NLCs were calculated after the determination of the entrapment rate, as described below.

### 2.3. Entrapment Efficiency, Size Determination and Morphology

To determine the percentage of Doxo entrapment in both Lipo- and NLC-formulations, Amicon^®^ Ultra 15 mL centrifugal filter units Millipore (Billerica, MA, USA) with a 100K cut-off were utilized. The liposome or NLC suspensions were subjected to centrifugation at 4600 rpm for 30 min at 25 °C in a Hettich Rotofix 46 centrifuge, and the retentate was collected. The process was repeated twice. Ethanol was partly evaporated during the preparation of liposomes and NLCs, and also together with the free doxorubicin, which was removed by ultrafiltration. After restoring the initial volume, the concentration of Doxo in the retentate was measured and compared to the initial concentration. Entrapment efficiency (EE%) was assessed using visible spectrophotometry by measuring the absorbance at 480 nm. For this purpose, liposomal or NLC suspensions were dissolved in a solution of 0.1% Triton X-100 in ethanol to ensure complete Doxo release. The EE% was calculated using the following equation: EE% = (Aret/Ain) × 100, where Ain is the absorbance of the initial suspension and Aret is the absorbance of the retentate suspension.

The particle sizes of all formulations were measured using laser diffraction with a Shimadzu SALD 2300 instrument (Shimadzu, Japan) operated via Wing SALDII software, version 3.4.10. The polydispersity index (PDI) was calculated in each sample to assess size distribution. Additionally, the morphology and particle size were examined using transmission electronic microscopy (TEM) with a Hitachi SU8320 FEG-STEM instrument, calibrated for a size range of 50 nm to 50 mm, to obtain TEM images.

### 2.4. Cell Cultures

The B16-F10 melanoma cell line, derived from mouse skin, was obtained from American Type Culture Collection (ATCC, Manassas, VA, USA) and cultured in Dulbecco’s Modified Eagle Medium (DMEM) supplemented with 10% fetal bovine serum (FBS), 1% L-glutamine, and 1% penicillin-streptomycin. Cells were maintained under standard conditions—95% relative humidity, at 37 °C, 5% CO_2_.

The Walker 256 cell line (also known as LLC-WRC 256), originally isolated from rat mammary carcinoma and adapted to an aggressive metastatic growth in suspension, was generously provided by Prof. Oliver Thews (Martin Luther University, Halle-Wittenberg, Germany). These cells were cultured in RPMI-1640 (Sigma-Aldrich, St. Louis, MO, USA) supplemented with 10% sterile fetal bovine serum, 20 mM HEPES, 10 mM L-glutamine, 10 mL of 7.5% NaHCO_3_, and 1 mL penicillin–streptomycin solution (Sigma-Aldrich; 10,000 units penicillin and 10 mg/mL streptomycin) per 100 mL of medium.

**Figure 1:**
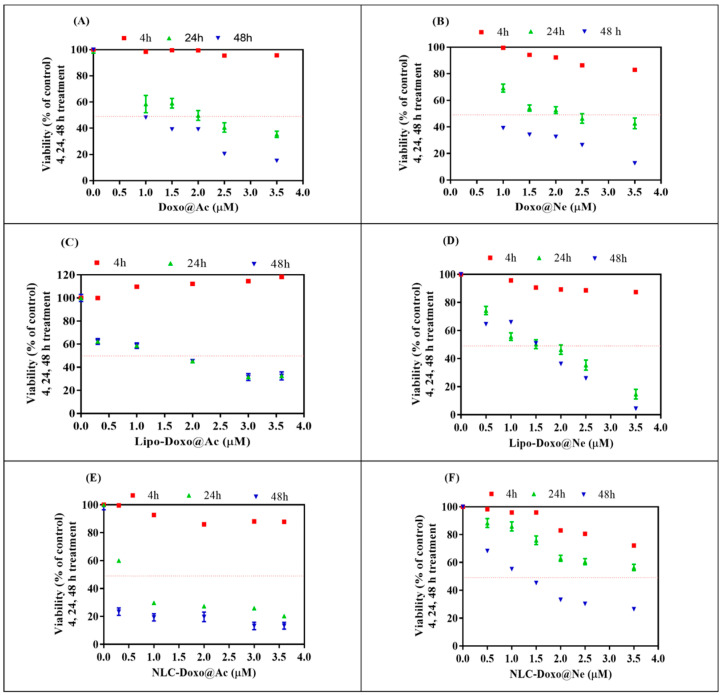
Viability of B16-F10 melanoma cells after the incubation (4, 24, 48 h) with successive concentrations (0.2–3.6 µM) of: Doxo@Ac (**A**); Doxo@Ne (**B**); Lipo-Doxo@Ac (**C**); Lipo-Doxo@Ne (**D**); NLC-Doxo@Ac (**E**); and NLC-Doxo@Ne (**F**).

**Figure 2:**
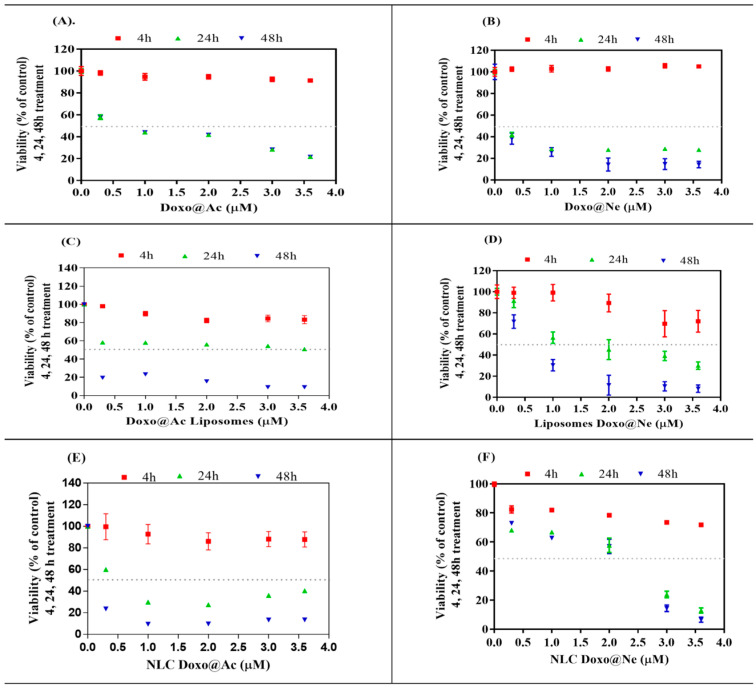
Viability of Walker 256 cancer cells after the incubation (4, 24, 48 h) with successive concentrations (0.2–3.6 µM) of: Doxo@Ac (**A**); Doxo@Ne (**B**); Lipo-Doxo@Ac (**C**); Lipo-Doxo@Ne (**D**); NLC-Doxo@Ac (**E**); and NLC-Doxo@Ne (**F**).

Both types of cell cultures were incubated in parallel with Doxo@Ac and Doxo@Ne stock solutions (40 µM) in ethanol:DMSO (3:1), diluted in culture media, and then added to the cell cultures to reach successive concentrations of 0.2 up to 3.5 or 3.6 µM, respectively, as shown in Figure 1 and Figure 2. The aqueous nanosuspensions of Lipo-Doxo@Ac, Lipo-Doxo@Ne, NLC-Doxo@Ac, and NLC-Doxo@Ne (having Doxo concentrations of 0.11–0.18 mM) were diluted in the same solvent mixture and subsequently added to the culture media to reach successive concentrations in the cell culture, from 0.2 up to 3.5 µM in the B16-F10 melanoma cell line, or 3.6 µM in the Walker 256 cell line, respectively, as shown in Figure 1 and Figure 2.

### 2.5. Viability and Cytotoxicity by MTT Assay

The MTT assay was used to evaluate the cytotoxic impact of Lipo-Doxo@Ac, Lipo-Doxo@Ne, NLC-Doxo@Ac, and NLC-Doxo@Ne comparative to Doxo@Ac and Doxo@Ne at similar concentrations, in the range 0.2–3.6 µM in B16-F10 melanoma and Walker 256 cell cultures. This assay is commonly used as a colorimetric test to determine cell cytotoxicity by providing information about cell proliferation, viability and toxicity. The 96-well plates were seeded with 8 × 103 B16-F10 cells and 1 × 104 Walker 256 cells, respectively, and kept for 24 h in the cell culture incubator; different concentrations of Doxo@Ac, Doxo@Ne, or Lipo-Doxo@Ac and Lipo-Doxo@Ne, or NLC-Doxo@Ac and NLC-Doxo@Ne, as mentioned above, were then added, incubated at 37 °C, and collected after 4, 24, and 48 h. After treatment, volumes of 10 µL MTT solution 12 mM in PBS were added to each well, followed by incubation at 37 °C for another 4 h. Subsequently, 150 µL of a DMSO: SDS 30% mixture (90:60, *v*/*v*) was added to each well. The plate was then shaken for 5 min at room temperature in the dark, followed by incubation for an additional 10 min at 37 °C. The absorbance of the resulting formazan product was measured at 550 nm using a microplate reader (Biotek, Synergy HT, CA, USA). Half-maximal inhibitory concentration (IC50) values were calculated applying the sigmoidal fitting equation. Statistical analysis was performed using one-way ANOVA followed by Tukey’s post hoc test using GraphPad prism software (version 6, GraphPad Software, CA, USA). Data are expressed as mean  ±  standard deviation (SD), with *n*  =  3, and a *p* ˂ 0.05 considered statistically significant.

### 2.6. Confocal Microscopy of Cells

For the morphological characterization of cells before and after the internalization of Doxo, the B16-F10 cells and Walker cells (35 × 10^3^ cells/well) were seeded on 2-well Lab-Tek chambered cover-glass and treated with Doxo@Ac (2 µM), Lipo-Doxo@Ac (2 µM) and NLC-Doxo@Ac (2 µM) for 24 h. The binding of Doxo to double-stranded DNA (dsDNA) was detected by visualizing the Doxo emission signal at 595 nm (red fluorescence) upon excitation with a 470 nm laser light. The cytoskeletal actin filaments of cells were stained with Phalloidin, CF^®^633, visualized by a green fluorescence (excitation/emission at 630/650 nm) using the excitation filter set, BP 470/40. Fluorescence images were acquired using a 40× objective and captured with an AxioCam MRc digital camera (Carl Zeiss AG, Oberkochen, Germany). Image processing was performed using Zeiss Zen 2.3 SP1 (Black Edition) software version 3.1 (Carl Zeiss AG, Oberkochen, Germany). The imaging was conducted on a Zeiss LSM 710 confocal laser scanning microscope (Carl Zeiss AG, Oberkochen, Germany) equipped with Argon and HeNe lasers, mounted on an Axio Observer Z1 Inverted Microscope (Carl Zeiss AG, Oberkochen, Germany).

### 2.7. Cell-Cycle Analysis by Flow Cytometry

The cell-cycle analysis and DNA content measurement of cells were assessed by flow cytometry using the fluorescent dye propidium iodide (PI), a membrane-impermeable fluorescent DNA stain used to mark cell death and also to differentiate apoptotic from necrotic cell death. For each cancer cell line, 1 × 10^6^ cells were seeded in 6-well plates and incubated for 24 h (B16-F10 melanoma adherent cell line) and 2 h (Walker 256 cell line in suspension), respectively, prior to treatment. Separate sets of treatments were prepared for the two cell lines using various concentrations of Doxo@Ac, Doxo@Ne, and the formulations Lipo-Doxo@Ac, Lipo-Doxo@Ne, NLC-Doxo@Ac, or NLC-Doxo@Ne. In each case, concentrations corresponding to the IC_50_ were used, as mentioned in Table 2. The cells were incubated for 24 h at 37 °C. After media removal, the adherent B16-F10 cells were detached with trypsin; their growth medium was also collected in a 15 mL tube and both were centrifuged for 3 min at 1500 rpm. The cell pellets from both cell lines were washed twice with cold PBS, and afterwards, 70% ethanol (2–5 mL) was added dropwise on ice. For fixation, both cell lines were kept at 4 °C for 30 min. Then, ethanol was removed by centrifugation (5 min, 2000 rpm), followed by washing twice with PBS. A volume of 50 μL RNAse (100 μg/mL) was added to each cell pellet, followed by incubation at 37 °C for 15 min. Finally, 200 μL of PI (50 μg/mL) was added; the cells were incubated for 10 min at 4 °C to achieve cell-cycle analysis. All data were collected, stored, and analyzed using a BD FACS Canto II flow cytometer (Becton Dickinson, Franklin Lakes, NJ, USA) and FACS Diva 6.1.3 software.

### 2.8. Apoptosis and Necrosis Assays

The percentage of cells undergoing apoptosis and/or necrosis was measured using the Annexin V-FITC and PI staining kit (Merck, Darmstadt, Germany) and analyzed using the same BD FACS cytometer. The analysis was performed on 1 × 10^6^ B16-F10 and Walker 256 cells, respectively, seeded in 6-well plates and incubated with Doxo@Ac, Doxo@Ne, and the formulations Lipo-Doxo@Ac, Lipo-Doxo@Ne, NLC-Doxo@Ac, or NLC-Doxo@Ne, at concentrations equivalent to their IC_50_, as mentioned above. After 24 h, wash steps of 3 min each with PBS were applied on the cells. Then, these were resuspended in 500 µL of binding buffer provided by the kit and transferred to flow cytometry tubes. Subsequently, 5 µL of Annexin V-FITC and 10 µL of propidium iodide (PI) solution were added to each sample. Following a 10 min incubation at room temperature in the dark, the cells were diluted twofold with cell wash solution. Data acquisition, storage, and analysis were performed using a BD FACS Canto II flow cytometer (Becton Dickinson, NJ, USA), equipped with FACS Diva 6.1.3 software.

The apoptosis and necrosis levels were established using the RealTime-Glo™ Annexin V Apoptosis and Necrosis Assay (Promega, Madison, WI, USA), a live-cell (non-lytic), real-time (kinetic) assay that measures the exposure of phosphatidylserine (PS) on the outer leaflet of the cell membrane during the apoptotic process. In the RealTime-Glo™ Assay, annexin V binding is detected by a luminescence signal, while necrosis is detected by a fluorescence signal. The assay signals were detected using a plate-based multimode reader, with fluorescence measured at 485 ± 20 nm excitation and collected at 525 ± 30 nm emission using a GloMax^®^ Discover (Promega) instrument.

### 2.9. Statistical Analysis

The statistical significance of the differences between groups was calculated using a one-way ANOVA. The significance of differences was established by using Dunnett’s multiple comparison test with cutoff values of *p* < 0.005 (***), *p* < 0.001 (**) and *p* < 0.05 (*).

## 3. Results and Discussion

### 3.1. Characterization of Doxorubicin Nanoformulations

The morphology of all formulations, as determined by TEM, is detailed in Supplementary File S1. The mean diameters (nm), polydispersity index (PDI), and entrapment efficiency (EE%) of the four nanoformulations (Lipo or NLC) containing Doxo@Ac or Doxo@Ne, compared to the controls, are presented in Table 1.

In this study, liposomes were prepared and used as a reference delivery system to evaluate the performance of NLCs, focusing specifically on the encapsulation and delivery potential for both acidic (Doxo@Ac) and neutral (Doxo@Ne) forms of doxorubicin.

As shown in Table 1, liposomes with both forms of doxorubicin exhibited smaller particle sizes overall (ranging from 295 to 409 nm). In contrast, NLCs displayed a wider range of sizes depending on the form of doxorubicin. Doxo@Ac-NLCs had the largest particles (459 ± 36.2 nm), while Doxo@Ne-NLCs were moderately smaller (335 ± 26.3 nm). This supports the hypothesis that NLCs are better suited for incorporating and stabilizing lipophilic drug molecules within their lipid-rich matrix. Furthermore, taking into consideration the values obtained from particle size measurements, we propose intratumoral administration as a viable further strategy, particularly for accessible tumor types, such as melanoma and breast cancer, which were further tested in this experiment. This localized delivery approach allows for the accumulation of therapeutic concentrations directly at the tumor site, potentially enhancing antitumor efficacy while minimizing systemic toxicity.

The polydispersity index (PDI) was low across all samples (<0.02), suggesting that the nanoparticle populations were uniform and stable, which is essential for controlled drug delivery.

The encapsulation efficiency (EE%) varied significantly between the formulations. As expected, liposomes achieved the highest EE% for Doxo@Ac (89.5 ± 3.55%), which aligns with the drug’s hydrophilic nature and its favorable partitioning in aqueous environments. Conversely, the EE% for Doxo@Ne in liposomes was lower (65.5 ± 5.06%) due to its lipophilic nature and reduced affinity for the aqueous core. In contrast, NLCs demonstrated improved encapsulation of Doxo@Ne (76.4 ± 4.86%) compared to Doxo@Ac (56 ± 3.75%).

The stability of all these formulations was checked during storage (one month at 4 °C), showing stable morphology and sizes. Additionally, smaller sizes (around 200–250 nm) were obtained by changing the ratio of Doxo@Ac and Doxo@Ne to lipid phases. However, these formulations showed lower entrapment efficiencies. Consequently, we selected formulations with larger sizes and a higher EE% for further measurements. These data are in good correlation with previous findings that compared nano-encapsulated hydrophilic Doxo@Ac and neutral Doxo@Ne [33]. However, limited studies have previously been performed to establish differences between these two chemical forms and to evaluate the impact of hydrophobicity on the anticancer activity of Doxo. Liposomes have proven to be good vehicles for delivering therapeutic agents into skin and cancer cells, being compatible and associating effectively with other hydrophobic lipid structures [7,19,20,21,22,24]. Few data were available about NLC co-loaded with Doxo as an anticancer, theragnostic agent in experimental models [35].

### 3.2. Cell Viability and Cytotoxicity

The MTT assay was used to evaluate the viability and cytotoxicity of melanoma B16-F10 and Walker 256 cells after treatment (4, 24, and 48 h) with different doses (range 0–3.6 µM) of Doxo@Ac or Doxo@Ne and the four Lipo- and NLC-formulations containing Doxo@Ac or Doxo@Ne (Figure 1 and Figure 2). The IC_50_ values (%) were calculated for each viability test and are presented in Table 2.

Free Doxo@Ac and Doxo@Ne both induced significant, time-dependent reductions in melanoma cell viability. After 24 h, Doxo@Ac had an IC_50_ of 1.82 µM and Doxo@Ne 2.18 µM, which dropped to 0.91 µM and 0.75 µM after 48 h, respectively. However, when encapsulated in liposomes, both forms showed enhanced cytotoxicity (IC_50_ ≈ 1.32 µM at 24 h), with no significant difference between the Doxo@Ac and Doxo@Ne formulations. In contrast, formulations based on NLC demonstrated a more varied cytotoxic profile. Among all the systems tested, NLC-Doxo@Ac was identified as the most potent, exhibiting the lowest IC_50_ value of 0.58 µM after 24 h (Figure 1E). On the other hand, the neutral form (NLC-Doxo@Ne) (Figure 1F) benefits from greater lipophilicity and compatibility with the matrix; however, its slower release kinetics or altered intracellular trafficking may lead to a more sustained but less immediate cytotoxic effect.

In Walker 256 cells, a more sensitive model, Doxo@Ne demonstrated stronger cytotoxic effects compared to Doxo@Ac, with IC_50_ values of 0.15 µM and 0.87 µM, respectively, at 24 h. The Lipo-Doxo@Ac system exhibited significant cytotoxicity with an IC_50_ of 0.37 µM, while Lipo-Doxo@Ne was less effective, showing an IC_50_ of 1.65 µM (Figure 2C,D; Table 2). Additionally, the NLC formulations maintained a dose-dependent inhibitory profile, with NLC-Doxo@Ac (IC_50_ = 0.76 µM) outperforming NLC-Doxo@Ne (IC_50_ = 1.35 µM) (Figure 2E,F; Table 2).

Interestingly, while Walker 256 cells generally showed greater sensitivity to the tested formulations, particularly those containing Doxo@Ac, an unexpected result emerged in the viability assays: lower IC_50_ values were observed in melanoma B16–F10 cells, especially for NLC-Doxo@Ac (IC_50_ of 0.58 µM after 24 h). We hypothesize that this apparent discrepancy may be due to cell line-specific differences in nanoparticle uptake or intracellular trafficking. Melanoma cells may have an enhanced capacity for endocytosis or possess membrane characteristics that facilitate the more efficient internalization of lipid-based nanocarriers, such as NLCs. This could lead to greater intracellular drug accumulation, despite these cells’ known resistance to treatment.

### 3.3. Internalization of Doxo@Ac Formulations in B16-F10 and Walker Cells

**Figure 3:**
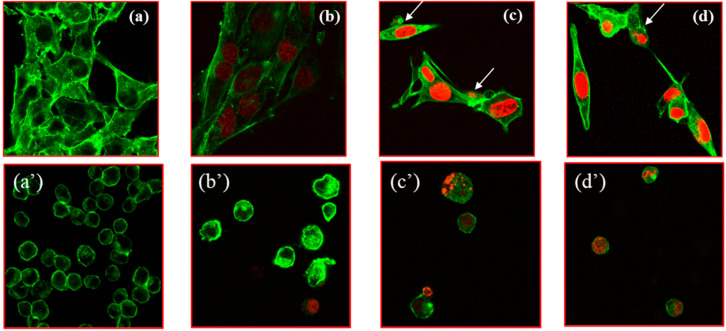
Confocal laser microscopic analysis of: non-treated B16-F10 cells (**a**); B16-F10 treated cells for 24 h with Doxo@Ac (red fluorescence) (2 µM) (**b**); Lipo-Doxo@Ac at 2 µM (**c**); NLC-Doxo@Ac (2 µM) (**d**); Walker 256 non-treated (**a’**); Walker 256 treated for 24 h with Doxo@Ac (red fluorescence) (2 µM) (**b’**); Lipo-Doxo@Ac at 2 µM (**c’**); and NLC-Doxo@Ac (2 µM) (**d’**). The cytoskeleton F-actin was stained with Phalloidin, CF^®^633 (green fluorescence). Magnification 40×.

Confocal laser microscopy was applied to check and confirm the previous findings in both cell cultures (Figure 3) by comparing the internalization rate of Lipo-Doxo@Ac and NLC-Doxo@Ac to free Doxo@Ac at a similar concentration (2 µM) after 24 h of incubation.

Compared to untreated cells (Figure 3a,a’), free Doxo@Ac was better internalized in melanoma cells (Figure 3b,b’). When comparing Doxo@Ac entrapped in Lipo vs. NLC, internalization was stronger in melanoma cells; intense chromatin condensation was observed as an indicator of apoptosis induction for Lipo-Doxo@Ac in both cell types. Nevertheless, despite these relevant images, it is difficult to quantitatively compare the degree of Doxo@Ac internalization in melanoma vs. Walker cells. Further studies provided complementary information, as described below.

### 3.4. Cell-Cycle Analysis

**Figure 4:**
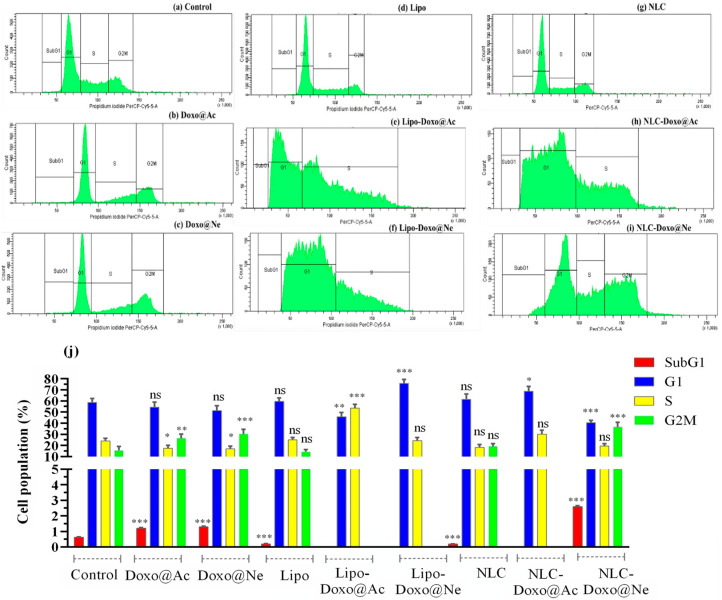
(**a**–**j**) Melanoma B16-F10 cells: cycle analysis by counting the distribution and arrest of the non-treated (control) B16-F10 cell population (**a**); B16-F10 cell population treated with Doxo@Ac (**b**); Lipo-Doxo@Ac (**c**); Lipo (**d**); Lipo-Doxo@Ac (**e**); Lipo-Doxo@Ne (**f**); NLC (**g**); NLC-Doxo@Ac (**h**); and NLC-Doxo@Ne (**i**).*, **, *** indicate *p* < 0.05, *p* < 0.01 and *p* < 0.001, respectively; ns means not significant.

**Figure 5:**
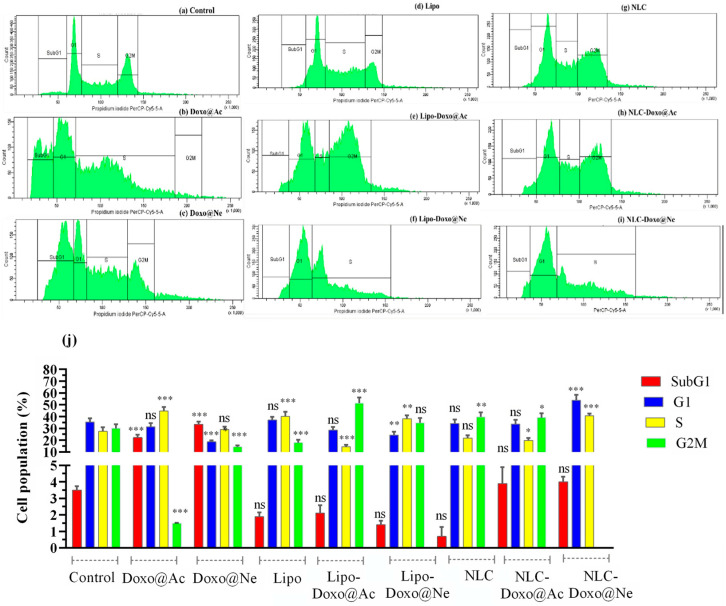
(**a**–**j**) **Walker 256 cells:** cycle analysis by counting distribution and arrest of non-treated (control) Walker 256 cell population (**a**); Walker 256 treated cell population with Doxo@Ac (**b**); Lipo-Doxo@Ac (**c**); Lipo (**d**); Lipo-Doxo@Ac (**e**); Lipo-Doxo@Ne (**f**); NLC (**g**); NLC-Doxo@Ac (**h**); and NLC-Doxo@Ne (**i**). *, **, *** indicate *p* < 0.05, *p* < 0.01 and *p* < 0.001, respectively; ns means not significant.

It is well documented that the impact of Doxo on viability and its cytotoxicity is due to cell-cycle arrest at different phases. After 24 h of incubation, using concentrations corresponding to the IC_50_ of each formulation (see Table 2), PI staining and flow cytometry analysis were used to assess cell-cycle distribution in melanoma B16–F10 and Walker 256 cell cultures (Figure 4 and Figure 5). The percentage of cell populations (by counting and distribution) and the phase-specific cell-cycle arrest (sub-G1, G1, S, and G2M) are represented.

No significant changes in viability were observed in melanoma cells in any cell-cycle phase when treated with Lipo and NLC (Figure 4d,g vs. Figure 4a) compared to untreated cells (controls). Doxo@Ac and Doxo@Ne slightly increased the percentage of cells in the G2M phase (Figure 4b,c). Notably, NLC-Doxo@Ac induced a significant accumulation of cells in the G1 phase, indicating effective cell-cycle arrest, while NLC-Doxo@Ne proved to be more apoptotic than Doxo@Ne and NLC-Doxo@Ac in the G2M phase. Significant modifications were observed in Lipo-Doxo@Ac and Lipo-Doxo@Ne compared to Doxo@Ac and Doxo@Ne (Figure 4e,f vs. Figure 4b,c). Lipo-Doxo@Ac induced cell-cycle arrest in G1 and S, while Lipo-Doxo@Ne was mostly active in G1, with no effects in the G2M phase. Figure 4j presents a comparative graph of these findings, showing the percentage of the cell population found in each cell-cycle phase.

In Walker 256 cells, an increase (though not statistically significant) in S phase arrest was observed when treated with Lipo and NLC compared to untreated cells (Figure 5d,g vs. Figure 5a). Among the various liposomal formulations, Lipo-Doxo@Ac was the most effective at inducing cell death, particularly through its accumulation in the S and G2/M phases of the cell cycle. In comparison, Lipo-Doxo@Ne exhibited a weaker pro-apoptotic effect (see Figure 5e,f). On the other hand, NLC-Doxo@Ac (see Figure 5h) demonstrated more balanced modulation of the cell cycle, with moderate increases observed in the G1 and S phases. This suggests a sustained antiproliferative action. Figure 5j presents a comparative graph of these findings, representing the cell population (%) found in each cell-cycle phase.

The comparative analysis of melanoma and Walker cells highlights the therapeutic potential of NLCs, particularly when loaded with Doxo@Ac. In melanoma cells, NLC-Doxo@Ac and both Lipo formulations primarily induced arrest in the early G1/S phase. For NLC, this pronounced effect may be attributed to a good internalization, which likely enhances intracellular drug accumulation. Importantly, this finding aligns with the viability assays, where NLC-Doxo@Ac exhibited the lowest IC_50_ values, reinforcing its potent cytotoxic activity in this cell line.

Although Walker 256 cells are intrinsically more sensitive to doxorubicin, as reflected by their higher apoptosis rates, they respond more strongly to liposomal formulations. More exactly, the Walker cells exhibited a pronounced response, showing greater cell-cycle arrest in both the S and G2/M phases, particularly after treatment with free doxorubicin and the liposomal formulations. Notably, the NLCs led to similar cell-cycle disturbances as the liposomes, with NLC-Doxo@Ac promoting cell accumulation in the G2/M phase. This suggests that this newer nanoparticle system is comparably effective and may offer advantages in drug delivery for solid tumors.

### 3.5. Apoptosis

**Figure 6:**
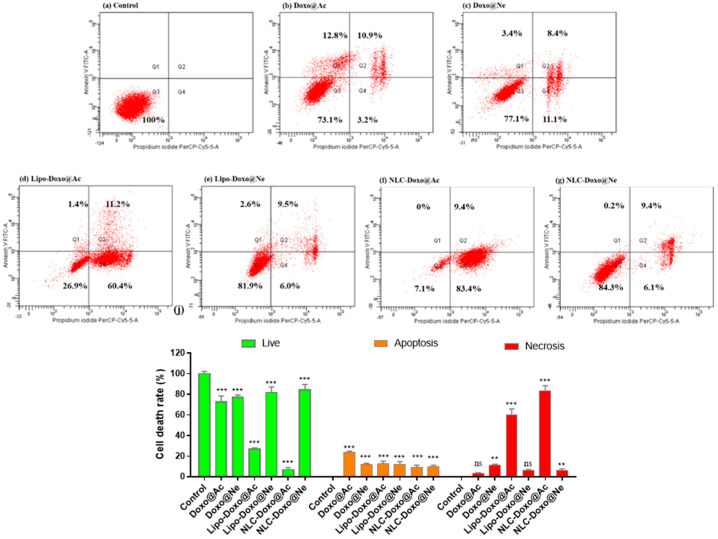
Flow cytometric analysis of Annexin V/PI-stained B16-F10 melanoma cells. Control (**a**); B16-F10 melanoma treated cells with Doxo@Ac (**b**); Doxo@Ne (**c**) Lipo-Doxo@Ac (**d**); Lipo-Doxo@Ne (**e**); NLC-Doxo@Ac (**f**); and NLC-Doxo@Ne (**g**). **, *** indicate *p* < 0.01 and *p* < 0.001, respectively; ns means not significant.

The progression of apoptosis was also monitored by flow cytometry using a standard apoptosis assay with PI and fluorescein isothiocyanate (FITC)-conjugated Annexin V (Annexin V-FITC). Late apoptosis or loss of membrane integrity in necrotic cells is marked by PI and, when combined with Annexin V, can distinguish different apoptotic stages. The results are presented below. Figure 6 includes the graphs for the melanoma cell analysis, from control (a) after treatments with Doxo@Ac, Doxo@Ne, and the formulations Lipo-Doxo@Ac, Lipo-Doxo@Ne, NLC-Doxo@Ac, and NLC-Doxo@Ne.

For Doxo@Ac-treated cells, 23.7% were found in apoptosis (12.8% in early and 10.9% in late apoptosis) (Figure 6b), whereas in Doxo@Ne-treated cells, 11.8% were found in apoptosis (3.4% in early and 8.4% in late apoptosis), with a higher percentage of necrotic cells (11.1%) (Figure 6c). Liposomal formulations of both Doxo forms triggered moderate apoptosis (~12%) (Figure 6d,e), but differed in their necrotic profiles, with Lipo-Doxo@Ac inducing a marked increase in necrotic cells (60.4%). Interestingly, NLC formulations, particularly NLC-Doxo@Ac, led to a dominant necrotic response (83.4%) with lower apoptotic induction. Although liposomes caused higher levels of apoptosis, NLCs exhibited distinct cytotoxic effects characterized by significant necrosis. In both types of formulations, the entrapment of Doxo@Ac resulted in a significant increase in apoptotic and necrotic cells. These data were positively correlated with the cell-cycle measurements (see Figure 4).

**Figure 7:**
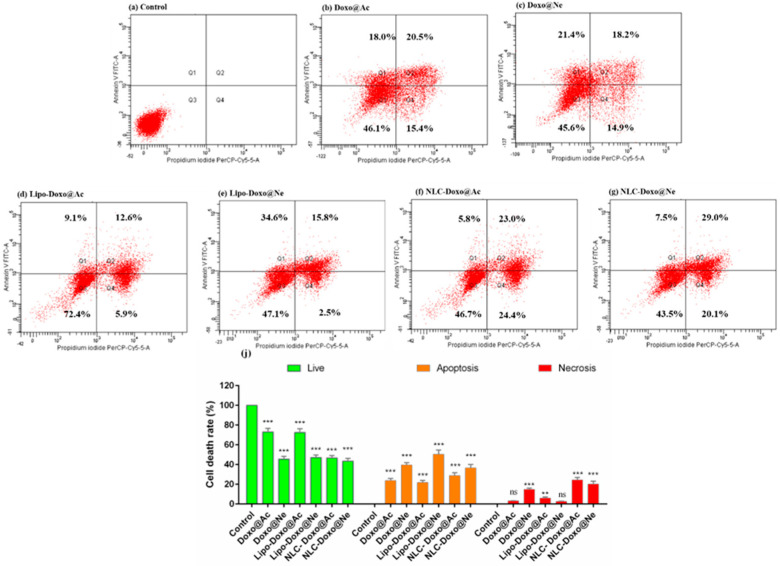
Flow cytometric analysis of Annexin V/PI-stained Walker 256 cells. Control (**a**); Walker 256 cells melanoma treated cells with Doxo@Ac (**b**); Doxo@Ne (**c**); Lipo-Doxo@Ac (**d**); Lipo-Doxo@Ne (**e**); NLC-Doxo@Ac (**f**); and NLC-Doxo@Ne (**g**). **, *** indicate *p* < 0.01 and *p* < 0.001, respectively; ns means not significant.

Figure 7 includes the graphs for Walker 256 cells analysis, from control (a) after treatments with Doxo@Ac, Doxo@Ne and formulations Lipo-Doxo@Ac, Lipo-Doxo@Ne, NLC-Doxo@Ac and NLC-Doxo@Ne.

In Walker 256 cells, for Doxo@Ac-treated cells, 38.5% of cells were found to be in apoptosis (18% in early and 20.5% in late apoptosis) (Figure 7b), whereas in Doxo@Ne-treated cells, 39.6% were found to be in apoptosis (21.4% in early and 18.2% in late apoptosis) (Figure 7c). The percentage of necrotic cells was similar (14.9% and 15.4%, respectively). Liposomal formulations displayed varying effects on apoptosis and necrosis; Lipo-Doxo@Ne resulted in the highest apoptosis rate at 50.4%, while Lipo-Doxo@Ac led to a lower apoptosis rate of 21.7%, accompanied by mild necrosis at 5.9% (Figure 7d,e). On the other hand, NLC-based formulations produced more balanced cytotoxic responses; NLC-Doxo@Ac induced 28.8% late apoptosis, while NLC-Doxo@Ne resulted in a higher late apoptosis rate of 36.5% (Figure 7f,g). Additionally, there was a significant increase in necrotic cells, particularly with NLC-Doxo@Ac, which showed a necrosis rate of 24.4%. These data were positively correlated with the cell-cycle measurements (see Figure 5).

The comparative analysis of apoptosis between melanoma B16-F10 cells and Walker 256 cells shows distinct responses to the different doxorubicin formulations tested. In melanoma cells, the formulation NLC-Doxo@Ac induced the most significant necrotic response, surpassing that of liposomal formulations. This finding is particularly important because melanoma cells often resist apoptosis due to intrinsic defects in apoptotic signaling; therefore, necrosis may serve as an alternative pathway for cell death. In contrast, Walker cells, which are generally more sensitive to drugs, exhibited the strongest apoptotic effect with the formulation Lipo-Doxo@Ne, followed closely by the NLC formulations. This suggests that, while both delivery systems are effective, the choice of formulation and the type of doxorubicin should be tailored to the tumor type’s sensitivity and preferred pathways for cell death. These data were positively correlated with the cell-cycle measurements (see Figure 5).

## 4. Conclusions

In conclusion, this study revealed distinct in vitro effects on B16-F10 melanoma cells and Walker 256 breast carcinoma cells when liposomal and nanostructured lipid nanoformulations with entrapped acidic, hydrophilic, and neutral, lipophilic Doxo were applied. More specifically, NLC-Doxo@Ac showed the best therapeutic effect in chemoresistant B16F10 melanoma cells, inducing strong cytotoxicity, necrosis, and G1/S phase arrest. By comparison, Walker 256 cells exhibited better sensitivity to the liposomal formulations; Lipo-Dox@Ac showed a strong cytotoxic effect and significant S/G2M arrest, whereas Lipo-Dox@Ne induced the highest apoptosis level.

All data reported here aim to fill an important gap in the literature by offering new insights into the distinct potential of NLC formulations to deliver the most efficient type of doxorubicin in accordance with the specific characteristics of each cancer type. Further studies aim to enhance NLC efficiency in Walker cells in vitro and in vivo by utilizing ultrasound-assisted delivery, with or without sonoporation, a minimally invasive theragnostic method, to improve its anticancer potential. Furthermore, the release kinetics of Doxo@Ne from NLCs may require further optimization, as they could underlie the lower-than-expected cytotoxic activity observed in both cancer models.

## Figures and Tables

**Table 1:** The mean diameters ± SD (nm) determined by SALD analysis, the PDI values, and the entrapment efficiency (EE%) were obtained from triplicate measurements of the four formulations: Lipo-Doxo@Ac, Lipo-Doxo@Ne, NLC-Doxo@Ac, and NLC-Doxo@Ne, compared to Lipo- and NLC (Controls). For details, see Methods.

Type	Parameters	Control	Doxo@Ac	Doxo@Ne
Lipo-formulation	Size (nm)	303 ± 20.20	295 ± 25.1	409 ± 45.25
	PDI	0.004	0.007	0.012
	EE%	-	89.5 ± 3.55	65.5 ± 5.06
NLC-formulation	Size (nm)	307 ± 35.15	459 ± 36.2	335 ± 26.3
	PDI	0.013	0.006	0.006
	EE%	-	56 ± 3.75	76 ± 4.86

**Table 2:** Comparative IC_50_ values (µM) of Doxo@Ac and Doxo@Ne delivered from Lipo (columns 3 and 4) and NLC (columns 5 and 6), versus free forms (Doxo@Ac and Doxo@Ne, columns 1 and 2), in melanoma B16-F10 (I) and Walker 256 cell cultures (II) after 24 h of incubation. The statistical significance threshold was set at *p* < 0.005.

Cell Line	Doxo@Ac	Doxo@Ne	Lipo-Doxo@Ac	Lipo- Doxo@Ne	NLC- Doxo@Ac	NLC- Doxo@Ne
I	1.82 ± 0.3	2.18 ± 0.5 ^ns^	1.33 ± 0.2 ^Ns, ####^	1.32 ± 0.1 ^Ns, ####^	0.58 ± 0.03 ^Ns, ####^	2.22 ± 0.9 ^Ns, NS^
II	0.87 ± 0.1	0.15 ± 0.03 ^ns^	0.37 ± 0.2 ^Ns, ####^	1.65 ± 0.04 ^Ns, ####^	0.76 ± 0.01 ^Ns, ##^	1.35 ± 0.02 ^Ns, ##^

^ns^ symbols were used to compare Doxo@Ac with Doxo@Ne. ^Ns^ symbols were used to compare Doxo@Ac with Lipo-Doxo@Ac/NLC-Doxo@Ac or Doxo@Ne with Lipo-Doxo@Ne/NLC-Doxo@Ne. ^##, ####, NS^ symbols were used to compare the controls (Lipo/NLC) with the nanoformulations.

## Data Availability

The authors confirm that the data supporting the findings of this study are available within the article and in its Supplementary Materials.

## Supplementary Materials

The following supporting information can be downloaded at: https://www.mdpi.com/article/10.3390/pharmaceutics17070904/s1.
