# Supplementary material for: Distinct In Vitro Effects of Liposomal and Nanostructured Lipid Nanoformulations with Entrapped Acidic and Neutral Doxorubicin on B16-F10 Melanoma and Walker 256 Carcinoma Cells
Source: Pharmaceutics. 2025 Jul 12;17(7):904. doi: 10.3390/pharmaceutics17070904 (PMC12299662; doi:10.3390/pharmaceutics17070904)

Supplementary file S1. Morphology of the Lipo- and NLC-nanoformulations , according to TEM at similar scales of 5 microns.

Lipo (Control)

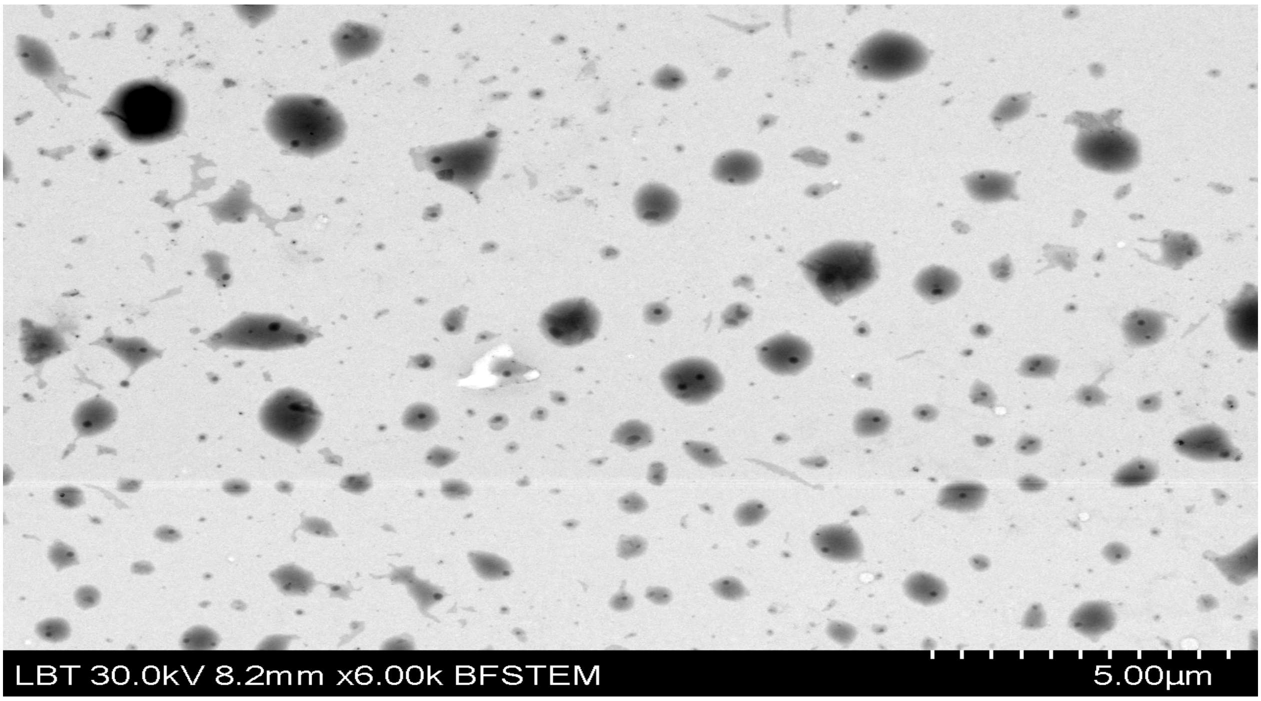

NLC (Control)

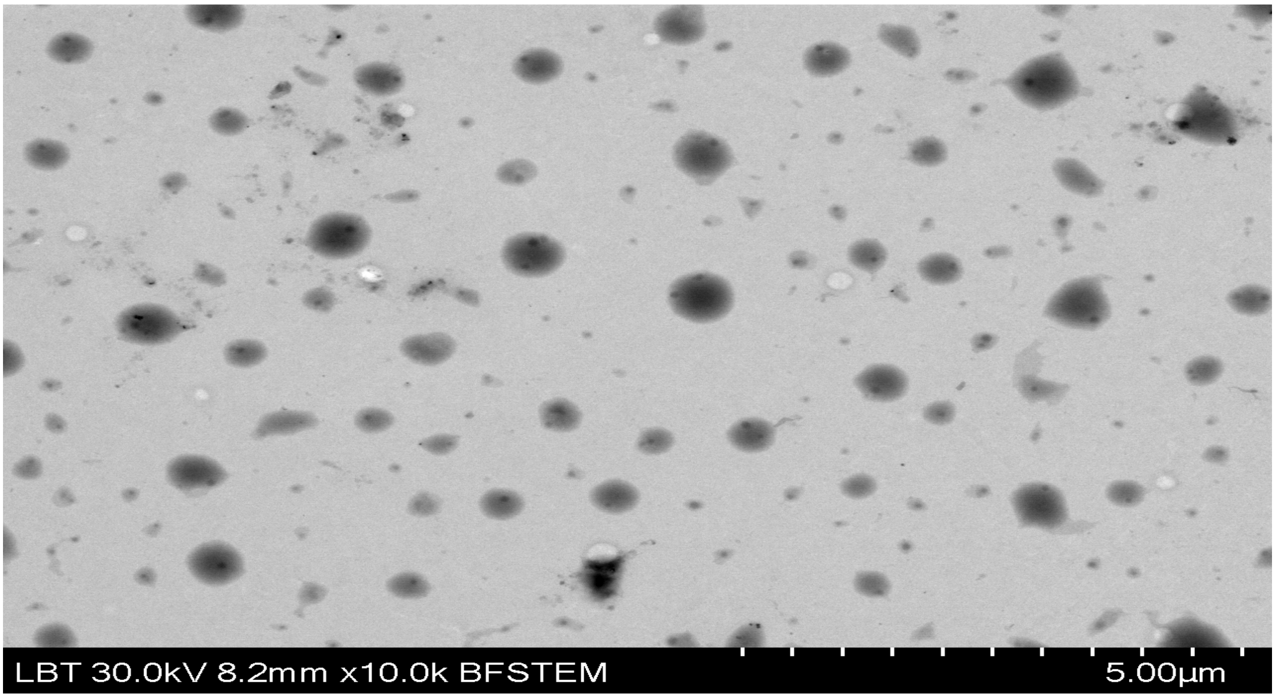

Lipo-Doxo@Ac

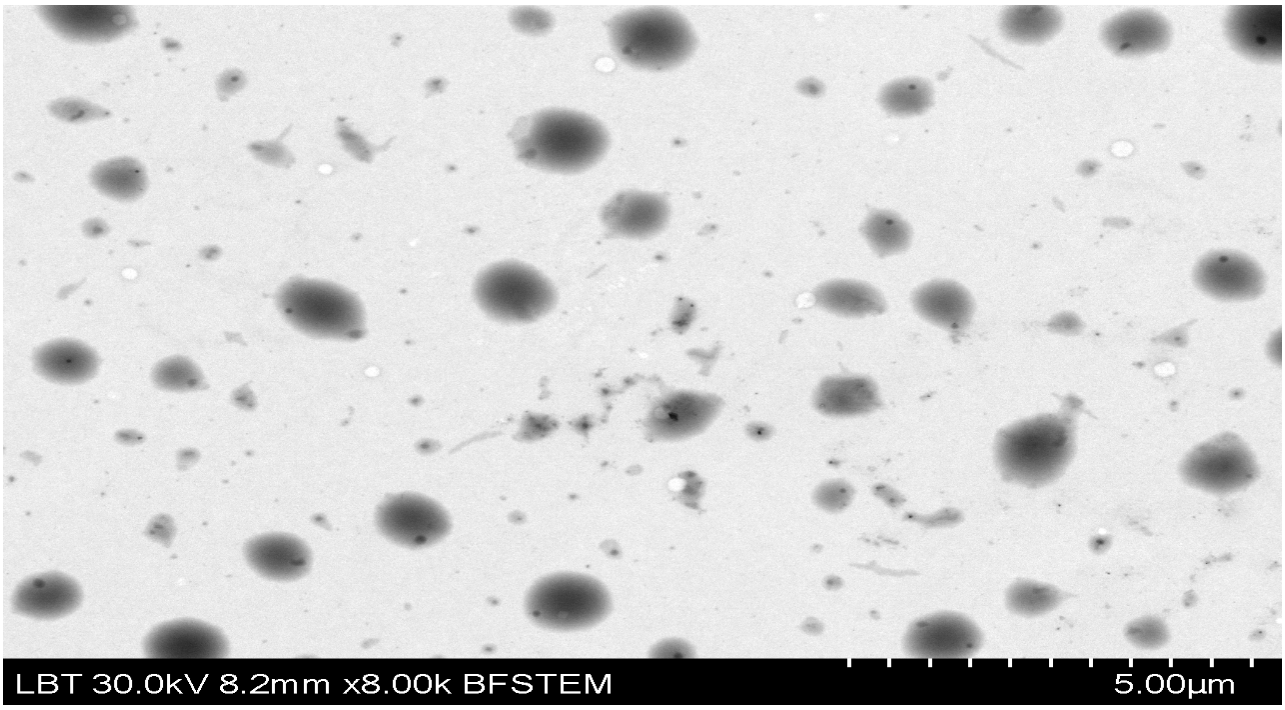

Lipo-Doxo@Ne

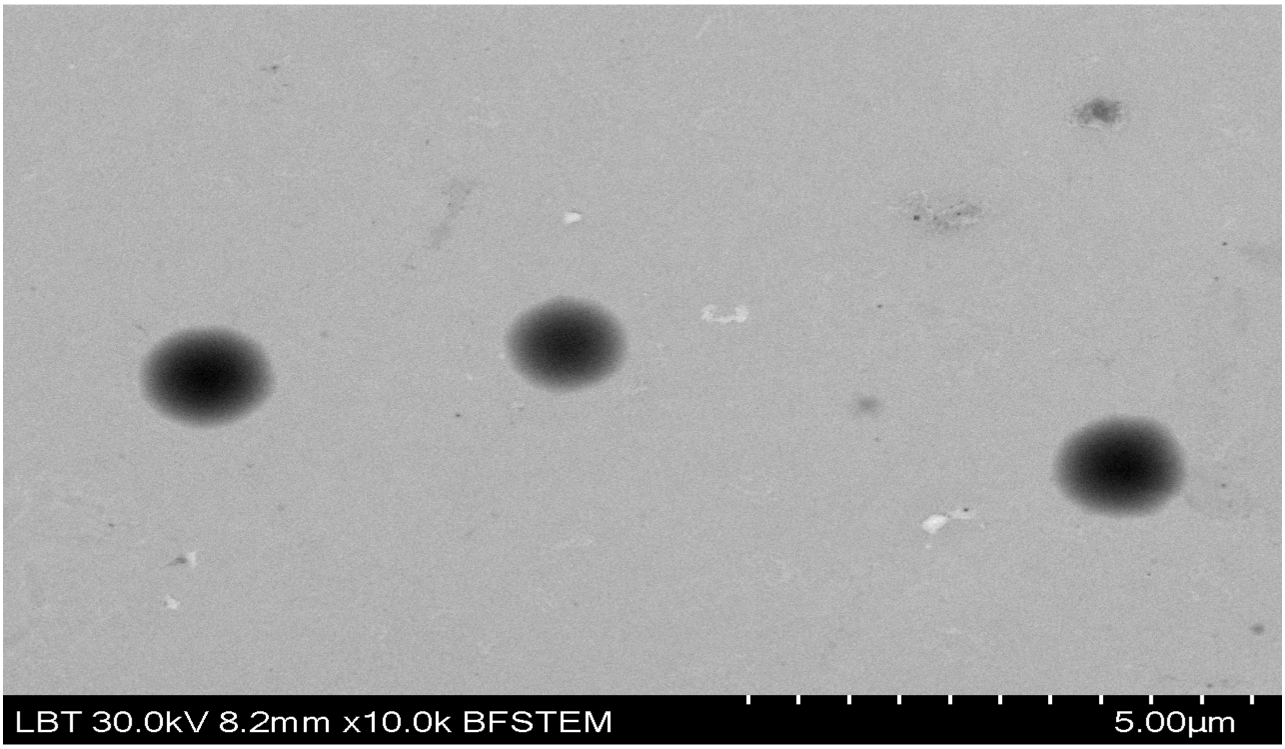

NLC-Doxo@Ac

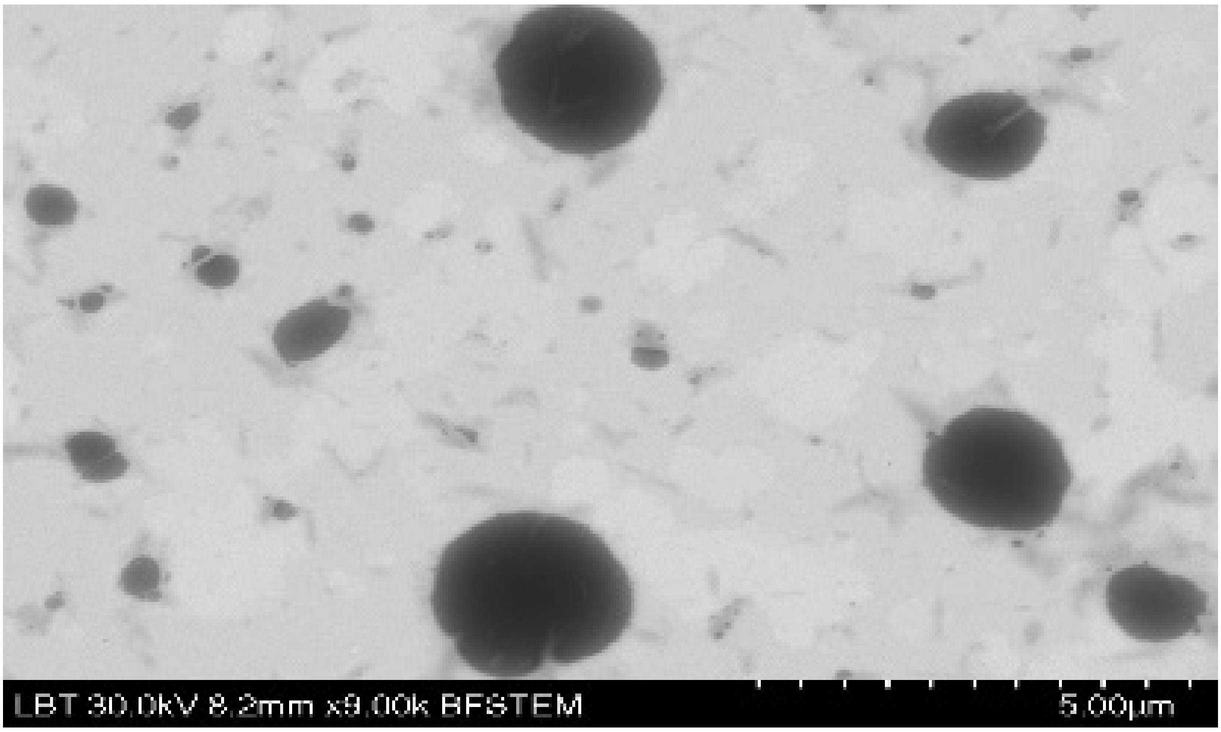

NLC-Doxo@Ne

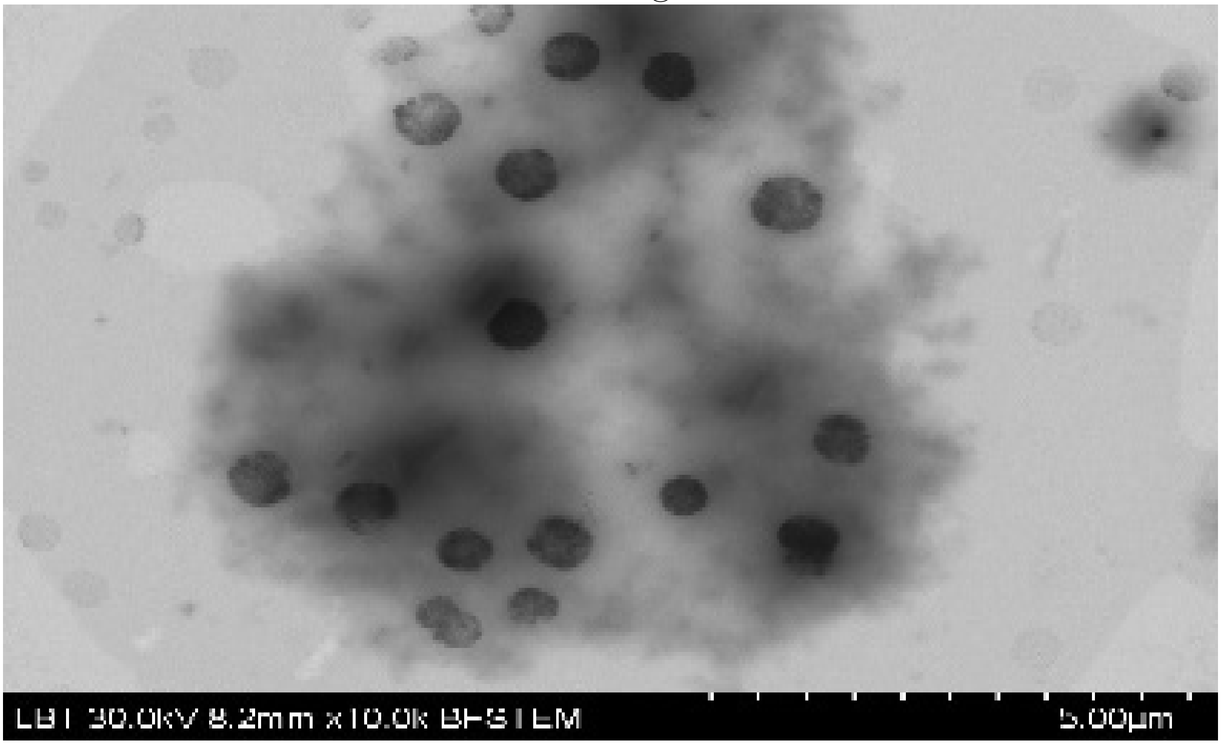

Supplement: Supplementary file 1 [file pharmaceutics-17-00904-s001.zip › pharmaceutics-3680170-supplementary.pdf]
